# Feasibility and robustness of dynamic ^18^F-FET PET based tracer kinetic models applied to patients with recurrent high-grade glioma prior to carbon ion irradiation

**DOI:** 10.1038/s41598-018-33034-5

**Published:** 2018-10-03

**Authors:** Charlotte Debus, Ali Afshar-Oromieh, Ralf Floca, Michael Ingrisch, Maximilian Knoll, Jürgen Debus, Uwe Haberkorn, Amir Abdollahi

**Affiliations:** 1 German Cancer Consortium (DKTK), Heidelberg, Germany; 2 Translational Radiation Oncology, National Center for Tumor Diseases (NCT), German Cancer Research Center (DKFZ), Heidelberg, Germany; 3 Division of Molecular and Translational Radiation Oncology, Heidelberg University Medical School, Heidelberg Institute of Radiation Oncology (HIRO), National Center for Radiation Research in Oncology (NCRO), Heidelberg, Germany; 4 Heidelberg Ion-Beam Therapy Center (HIT), Department of Radiation Oncology, Heidelberg University Hospital, Heidelberg, Germany; 5 Department of Nuclear Medicine, Heidelberg University Hospital, Heidelberg, Germany; 6 Clinical Cooperation Unit Nuclear Medicine, German Cancer Research Center (DKFZ), Heidelberg, Germany; 7 Department of Nuclear Medicine, Inselspital, Bern University Hospital, University of Bern, Bern, Switzerland; 8 Division of Medical Image Computing, German Cancer Research Center (DKFZ), Heidelberg, Germany; 9 Department of Radiology, University Hospital Munich, Ludwig-Maximilians-University Munich, Munich, Germany

## Abstract

The aim of this study was to analyze the robustness and diagnostic value of different compartment models for dynamic ^18^F-FET PET in recurrent high-grade glioma (HGG). Dynamic ^18^F-FET PET data of patients with recurrent WHO grade III (n:7) and WHO grade IV (n: 9) tumors undergoing re-irradiation with carbon ions were analyzed by voxelwise fitting of the time-activity curves with a simplified and an extended one-tissue compartment model (1TCM) and a two-tissue compartment model (2TCM), respectively. A simulation study was conducted to assess robustness and precision of the 2TCM. Parameter maps showed enhanced detail on tumor substructure. Neglecting the blood volume V_B_ in the 1TCM yields insufficient results. Parameter K_1_ from both 1TCM and 2TCM showed correlation with overall patient survival after carbon ion irradiation (p = 0.043 and 0.036, respectively). The 2TCM yields realistic estimates for tumor blood volume, which was found to be significantly higher in WHO IV compared to WHO III (p = 0.031). Simulations on the 2TCM showed that K_1_ yields good accuracy and robustness while k_2_ showed lowest stability of all parameters. The 1TCM provides the best compromise between parameter stability and model accuracy; however application of the 2TCM is still feasible and provides a more accurate representation of tracer-kinetics at the cost of reduced robustness. Detailed tracer kinetic analysis of ^18^F-FET PET with compartment models holds valuable information on tumor substructures and provides additional diagnostic and prognostic value.

## Introduction

Despite extensive research, prognosis of patients diagnosed with high-grade glioma (HGG) remains poor^1–3^. Persistent remissions are rare and local tumor progress still poses a major pattern of therapy failure^4,5^.

Biological imaging, such as positron emission tomography (PET), can be used to assess tissue metabolism and represents a promising modality to assist in diagnosis and planning of the treatment^6^.

In recent years, ^18^F-labeled fluoro-ethyl-tyrosine (FET)^7^ has been increasingly used for imaging HGG^8–13^. ^18^F-FET has been shown to provide high sensitivity and specificity of the tracer for glioma tissue and at the same time low uptake in inflammatory and healthy brain tissue^14^. The high *in-vivo* stability of FET and long half-life of ^18^F, together with the easy production in sufficient amounts, make the tracer practical for clinical applications. The role of ^18^F-FET in both diagnosis as well as therapy monitoring of HGG has been intensively studied^8,15,16^, proving the value of ^18^F-FET for lesion detection in low- and high-grade gliomas as well as the identification of tumor recurrence. Tracer uptake in terms of the standard uptake value (SUV) has further been correlated with tumor cell density^17^.

However, it has been shown that the amount of tracer uptake and the subsequent correlation of the SUV with tumor grading depend on the acquisition timing following tracer injection^18^. Hence, static PET scan protocols might not reveal the entire metabolic tumor structure, and results can vary for different acquisition schemes.

Advanced pharmacokinetic analysis of time-activity curves (TACs) from dynamic PET scans using compartment models enables for the extraction of direct physiological correlates and helps in the comprehension of the underlying fundamental uptake mechanisms^19^. This is of special interest, as it may allow the assessment of tumor physiology and eventual heterogeneity that might not be visible with standard modalities, like magnetic resonance imaging (MRI) or computed tomography (CT).

^18^F-FET, unlike other system L- substrates like [^11^C] methionine and [^18^F]FDOPA, is not further metabolized after being taken up by the cells, which simplifies respective compartment models and thus makes it a potentially valuable candidate for tracer kinetic analysis. Several authors have studied the value of dynamic ^18^F-FET PET for diagnosis and grading of HGG^9,20–22^. However, most of them resort to either qualitative analysis of curve shapes or derivation of semi-quantitative parameters such as the initial slope or time to peak. Few authors applied detailed pharmacokinetic analysis using different compartment models^23,24^, but parameter robustness and reliability were not investigated.

The aim of the present study was to investigate feasibility of tracer kinetic analysis in dynamic ^18^F-FET PET of recurrent HGG and to identify the most accurate and reliable model. Therefore, we systematically investigated fits with the 1TCM, the simplified 1TCM and the 2TCM to data from patients with recurrent HGG. Quality of the 2TCM fits was assessed by fitting synthetic TACs, generated with different parameter combinations, and analyzing the resulting parameter estimates in terms of accuracy and robustness.

## Methods

All procedures performed in this study were in accordance with the 1964 Helsinki declaration and its later amendments or comparable ethical standards. The study was approved by the institutional ethics committee (ethics committee of the University of Heidelberg, S-421/2015). Owing to the retrospective nature, the need for informed consent was waived.

### Compartment models

**Figure 1:**
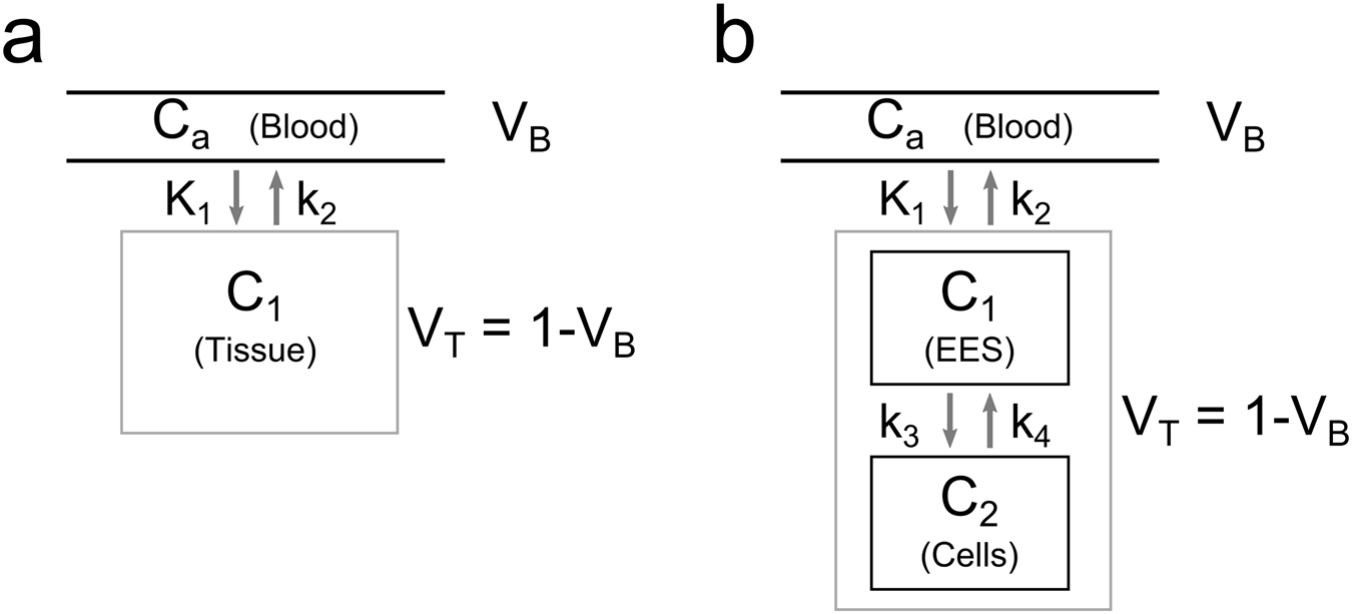
Schematic representation of the used compartment models. C_a_ is the concentration of tracer in the arterial blood (arterial input function AIF). (**a**) The one-tissue compartment model (1TCM) describes the exchange of tracer between the arterial blood with the tissue in terms of exchange rates K_1_(1TCM) and k_2_(1TCM). In an element of tissue the blood compartment in form of the capillaries occupies the volume fraction V_B_, whereas the fraction of tissue volume is V_T_ = 1-V_B_. (**b**) The more complex two-tissue compartment model (2TCM) separates the tracer concentration in tissue into tracer in the extracellular extravascular space (EES) and in the cells. Tracer is transported from the capillaries into the EES over the blood-brain barrier with rates K_1_(2TCM), k_2_(2TCM), and from the EES it is taken up in the cells with rates k_3_(2TCM), k_4_(2TCM).

Different compartment models could be feasible to describe ^18^F-FET tracer kinetics. The simplest approach is using a standard one-tissue compartment model (1TCM), which is depicted in Fig. 1a. The exchange of tracer between blood and tissue by means of exchange rate constants $[{K}_{1},{k}_{2}]$ can be expressed in a single differential mass balance equation:

1 $$\frac{d{C}_{t}(t)}{dt}={K}_{1}{C}_{a}(t)-{k}_{2}{C}_{t}(t)$$

${C}_{a}(t)$ and ${C}_{t}(t)$ represent the tracer concentrations within the arterial blood pool and tissue, respectively.

The total measured concentration of tracer in a volume element then yields:

2 $${C}_{T}(t)=(1-{V}_{B}){C}_{t}(t)+{V}_{B}{C}_{a}(t)$$

${V}_{B}$ represents the relative volume of blood within in a respective volume element (in ml/100 ml tissue, $\,0 < {V}_{B} < 1$)^19^. In many cases, ${V}_{B}$ is assumed to be zero, leading to a simplified model with only two parameters ${K}_{1}\,\,$and ${k}_{2}$^25^. In the following, this model will be referred to as “simplified 1TCM” (s1TCM). The two transfer constants [${K}_{1},\,{k}_{2}$] in the 1TCM include both tracer transport over the blood-brain barrier via blood flow and diffusion and transport of the tracer to the cells. After crossing the blood-brain barrier, ^18^F-FET is transported into the cells by the LAT system^26^. Hence, a more detailed description of the transport processes could be formulated in a two-tissue compartment model (2TCM)^19^, which is described by two differential equations with transfer constants [${K}_{1},\,{k}_{2},\,{k}_{3},\,{k}_{4}$]:

3 $$\frac{d{C}_{1}(t)}{dt}={K}_{1}{C}_{a}(t)-({k}_{2}+{k}_{3}){C}_{1}(t)+{k}_{4}{C}_{2}(t)$$

4 $$\frac{d{C}_{2}(t)}{dt}={k}_{3}{C}_{1}(t)-{k}_{4}{C}_{2}(t)$$

The interplay of the two tissue compartments is illustrated in Fig. 1b. *C*_1_(*t*) represents the concentration of tracer in the interstitial space, whereas *C*_2_(*t*) is the concentration within cells. Transport rates ${K}_{1}\,\,$and ${k}_{2}$ describe blood flow and diffusion of the tracer into the interstitial space, and ${k}_{3}$ and $\,{k}_{4}$ the transport via the LAT system.

The total tracer concentration *C*_*T*_(*t*) in a voxel of tissue corresponds to the time-activity curve (TAC) derived from dynamic PET measurements. The concentration in the arterial blood pool is usually derived from the 4D PET images by extracting the TAC in a feeding artery close to the tissue of interest. This arterial TAC is often referred to as arterial input function (AIF). For tracer-kinetic analysis, the functional representation of *C*_*T*_(*t*) from the respective model is fitted to the measured TAC in tissue by means of non-linear least square techniques, using the measured arterial concentration. The fitting routine in terms yields estimates for the models transfer constants.

The subsequent bidirectional transport of ^18^F-FET into and out of the cells after crossing the BBB can be modeled by application of a 2TCM. However, if data quality is low, e.g. due to low temporal resolution or low signal-to-noise ratio (SNR), a simplified one tissue compartment model (1TCM) could be more suitable as it yields more stable parameter estimates and does not pose the risk of overfitting the data.

### Patient data

16 patients with recurrent HGG were investigated in this institutional review board approved retrospective analysis. Patient characteristics are listed in Table 1. Tumor grade was evaluated at diagnosis of tumor recurrence based on clinical features on MRI.

**Table 1:** Patient Characteristics.

Feature			Grade III	Grade IV
	Gender	Male	6	6
		Female	1	3
Primary Treatment	Resection	Complete	1	2
	(surgery)	Partial	2	6
		Biopsy only	4	1
	Chemotherapy	TMZ	5	9
	Radiotherapy	Photon	6	9
		Ion	1	0
At time of recurrence diagnosis	Age	<50	1	4
		50–59	4	1
		60–69	1	3
		≥70	1	1
	KPS	100	1	4
		90	5	3
		≤80	1	1
		NA	0	1
After Re-irradiation	Status at last FU	PD	5	7
		SD	2	2
	Death		4	8

^18^F-FET PET scans were acquired prior to re-irradiation using a Siemens Biograph 6 PET/CT scanner. Median injected activity was 190 MBq (130 to 235 MBq). Dynamic acquisition was performed over 40 min in 20 frames with temporal sampling of 6 × 20 s, 8 × 60 s and 5 × 300 s. Endpoint static ^18^F-FET PET images were calculated by averaging the scans of the last 10 minutes. Images featured a resolution of 1.33 mm × 1.33 mm × 3 mm, acquired over 80 slices. Static ^18^F-FET PET raw images were converted to SUV images, and tracer-enhancing tumor lesions were segmented based on an isocontour of 70% (I_70_). Various thresholds are in practice for isocontours, ranging from 50% to 90%. ^18^F-FET uptake is low compared to other tracers like FDG. Therefore, to our experience in recurrent glioma, I_90_ yields very small volumes of only a few voxels. I_50_ on the other hand can easily result in wide spreading segmentations of half of the brain, as recently shown^27^. Thus, I_70_ was selected as best compromise between these values.

SUV_max_ was determined as maximum value within I_70_. The *standardized uptake ratio* (SUR) was calculated by normalizing SUV_max_ to the mean background uptake (SUV_bg_), which was derived as average SUV within a region of similar 2D size in a part of the brain contralateral to the tumor.

Patient individual image-derived arterial TACs were extracted from the left/right carotid artery in three image slices. Segmentations of arterial TACs were delineated based on the first and second time frame and chosen to include only the inner part of the artery by selection of voxels with maximum peak activity (visual inspection of voxel-wise TACs) and by co-registering contrast enhanced MRI, in order to minimize partial volume effects. Whole blood to plasma conversion was applied using a factor of 10%.

Voxelwise fitting of TACs with 1TCM, s1TCM and 2TCM was performed within I_70_, yielding parameter estimates K_1_ (s1TCM), k_2_ (s1TCM), K_1_ (1TCM), k_2_ (1TCM), V_B_ (1TCM), K_1_ (2TCM), k_2_ (2TCM), k_3_ (2TCM), k_4_ (2TCM), V_B_ (2TCM). For all transfer rates a constraint of 0 min^−1^ < *k*_*i *_< 1 min^−1^ was set in order to limit the search space and exclude unreasonable values. The maximum value of respective parameter estimates was defined as the 75^th^ quantile, since the actual maximum value potentially consists of the set parameter constraints. Median and maximum of each of the 6 parameters were correlated with tumor grade. Tumors were irradiated with fractionated carbon ions at a median total dose of 37.5 GyE (range 30 to 42 GyE, 3 GyE per fraction). Median parameter estimates of the 1TCM and 2TCM as well as SUR and grade were correlated with overall survival (OS) and progression free survival (PFS) after re-irradiation using Cox proportional hazards model.

### Simulation Data

For assessment of stability and precision of the parameter estimates resulting from fits with the 2TCM, synthetic TACs were generated using various parameter combinations of K_1_, k_2_, k_3_, k_4_ and V_B_ (2TCM). To our knowledge, no detailed reports on tracer kinetic modeling in dynamic ^18^F-FET PET in recurrent HGG exist. Thus, representative values of the model parameters, which could be used for data simulation, were not available. Representative sets of parameters for the 2TCM were derived from patient parameter estimates of the current analysis. Histograms of parameter distributions of each patient were investigated and peak values were extracted. From these, ten common parameter combinations were derived (see results). Using the parameter combinations and two different measured patient AIFs, 1000 TACs were generated for each parameter combination using the 2TCM model function.

Acquisition noise was simulated by adding Gaussian random numbers to the synthetic curves, resulting in a signal-to-noise ratio (peak of AIF, divided by standard deviation of the noise) of SNR = 100. Simulated TACs were fitted with the 2TCM, using the same AIF as for data generation.

### Evaluation of parameter estimates

Quality of fits was evaluated by means of reduced ${{\chi }^{2}}_{red}$, calculated as the ratio between ${\chi }^{2}\,\,$and the number of degrees of freedom f:

5 $${{\chi }^{2}}_{red}=\frac{1}{f}\cdot \frac{1}{{\rm{\Delta }}{y}^{2}}\cdot SSR,\,\,\,\,\,\,\,\,f=n-p,\,$$

with n being the number of sampling steps, p the number of model parameters, and SSR the sum-of-squared-residuals. ${\rm{\Delta }}{y}^{2}$ is the squared error on each measured activity value y. For real patient data, the exact error on the measured activity is usually not known, which complicates calculation of ${{\chi }^{2}}_{red}$. However, apart from systematic errors of the acquisition scanner that are identical for all measurements, the error on the measured activity y is influenced by the statistical nature of the radioactive decay. Therefore, we assumed ${\rm{\Delta }}y\approx \,\sqrt{y}$ . For simulated curves, the noise level is known, thus the error on the measured activity can be simply derived as ${\rm{\Delta }}y={\sigma }_{Noise}=\frac{AI{F}_{peak}}{CNR}$.

${{\chi }^{2}}_{red}$ is mainly driven by the SSR, indicating a better fit quality if the model curve lies closer to the measured data points. However, the more parameters a model provides, the better it can approximate the measured data, regardless of validity of these parameters. Hence, the Akaike information criterion (AIC)^28^ provides a more reliable quantity for fit evaluation, as it penalizes the fit quality of a model with the number of parameters^29^:

6 $$AIC=N\cdot \,\mathrm{ln}(\frac{SSR}{n})+2(p+1)$$

The corrected AIC (cAIC) for small sample sizes is calculated as:

7 $$cAIC=AIC+\frac{2(p+1)(p+2)}{n-p-2}$$

When comparing fits from different models, the model yielding the lowest cAIC is considered to yield the best representation of the measured data. For comparison of the three models used in this study, the cAIC was computed for each fit, and results among models were compared.

Accuracy and stability of parameter estimates from fitting the simulated TACs (${P}_{fit}$) were evaluated in terms of the absolute and relative deviation from the true value (${P}_{input}$), referred to as absolute and relative error:

8 $${E}_{abs}={P}_{fit}-{P}_{input}$$

9 $${E}_{rel}=\frac{{P}_{fit}-{P}_{input}}{{P}_{input}}\cdot 100 \% $$

### Software

All image analysis was conducted using the Medical Imaging interaction ToolKit (MITK)^30^. SUV calculations were conducted using the SUV calculation plugin. For segmentation of tumor lesions, the MITK isocontour plugin^31^ was used. Tracer kinetic analysis was performed using an in-house developed software module for pharmacokinetic modeling in MITK^32^, which allows for voxelwise fitting of the measured time activity curves with various compartment models. The tool is incorporated into MITK and applies a Levenberg-Marquardt algorithm for model fitting.

For statistical analysis and plotting the open-source *R* software package was utilized (version 3.3.2, http://www.R-project.org). Two-sided, unpaired Wilcoxon tests were applied, together with Bonferroni-Holmes multiple testing corrections. Mantel-Cox log-rank test was used to compare differences in Kaplan-Meier survival curves (KM). Cox proportional hazards model was used for univariate analyses using log-transformed median parameter values (survival package, version 2.40-1, https://CRAN.R-project.org/package=survival). The significance level was set to 0.05.

## Results

Regions of increased tracer uptake were delineated on basis of endpoint ^18^F-FET PET images using an isocontour of 70% (I_70_) and SUV_max_ and SUR were calculated. Within the isocontour, voxelwise tracer kinetic analysis was conducted using the simplified 1TCM (without blood volume V_B_), the standard 1TCM and 2TCM (both with blood volume).

**Figure 2:**
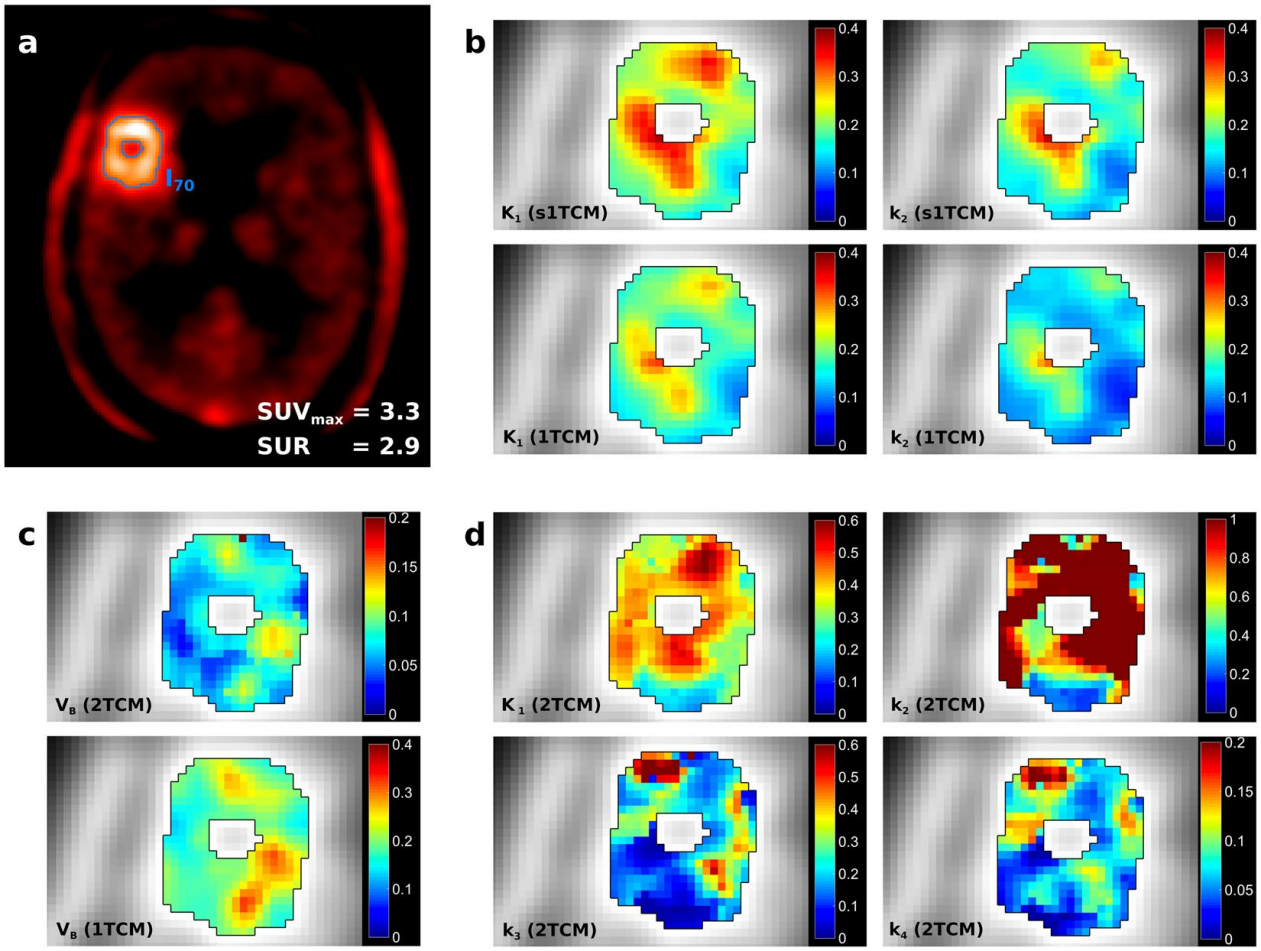
Parameter maps from fits with different compartment models in an exemplary patient with grade IV glioma. Pharmacokinetic analysis was performed within an isocontour of 70%, which was delineated based on the endpoint static summation image, as illustrated in (**a**). SUV_max_ and SUR were extracted, where SUR is the ratio between SUV_max_ and mean background SUV. Both the standard 1TCM and a simplified 1TCM were fitted voxelwise to the time-activity curves (TACs), yielding estimates for exchange rates K_1_ and k_2_ (**b**). Furthermore, TACs were fitted with the more complex 2TCM, which yields metabolic exchange rates K_1_, k_2_, k_3_ and k_4_ (**d**). The general definitions of the 1TCM and 2TCM yield estimates on the blood volume V_B_, which is displayed in (**c**). In the simplified 1TCM (s1TCM), the blood volume is assumed to be zero V_B_ = 0. Parameter maps of tracer kinetic analysis show enhanced detail on tumor substructure, however, stability is decreased for the 2TCM. Both 1TCM and 2TCM yield similar distributions of V_B_, indicating that the parameter is not negligible.

Figure 2 shows corresponding parameter maps from fits with the different models in an exemplary patient. K_1_ and k_2_ from the simplified and standard 1TCM (Fig. 2b) showed tumor substructure in more detail compared to the static SUV image. Parameter maps of K_1_ and k_2_ are very similar, with hot-spots and cold-spots in the same areas. However, the simplified 1TCM yields higher values in both parameters.

Fits with the 2TCM (Fig. 2d) could resolve the substructure even further; however, the enhanced level of detail of the 2TCM also resulted in less stable parameter estimates. This is visible in k_2_, where dark-red voxels indicate misfits, which approached the parameter constraint boundaries. Parameter maps of k_3_ and k_4_ present with congruent distributions, however, values of k_3_ are higher in hotspots. V_B_ from the 1TCM and 2TCM show also very similar distributions (Fig. 2c), indicating stability and consistency of the parameter over the models. Estimates for V_B_ are higher from 1TCM compared to 2TCM. These results indicate that the blood volume V_B_ is not negligible in tracer kinetic analysis of dynamic ^18^F-FET PET.

### Evaluation of fit quality

**Figure 3:**
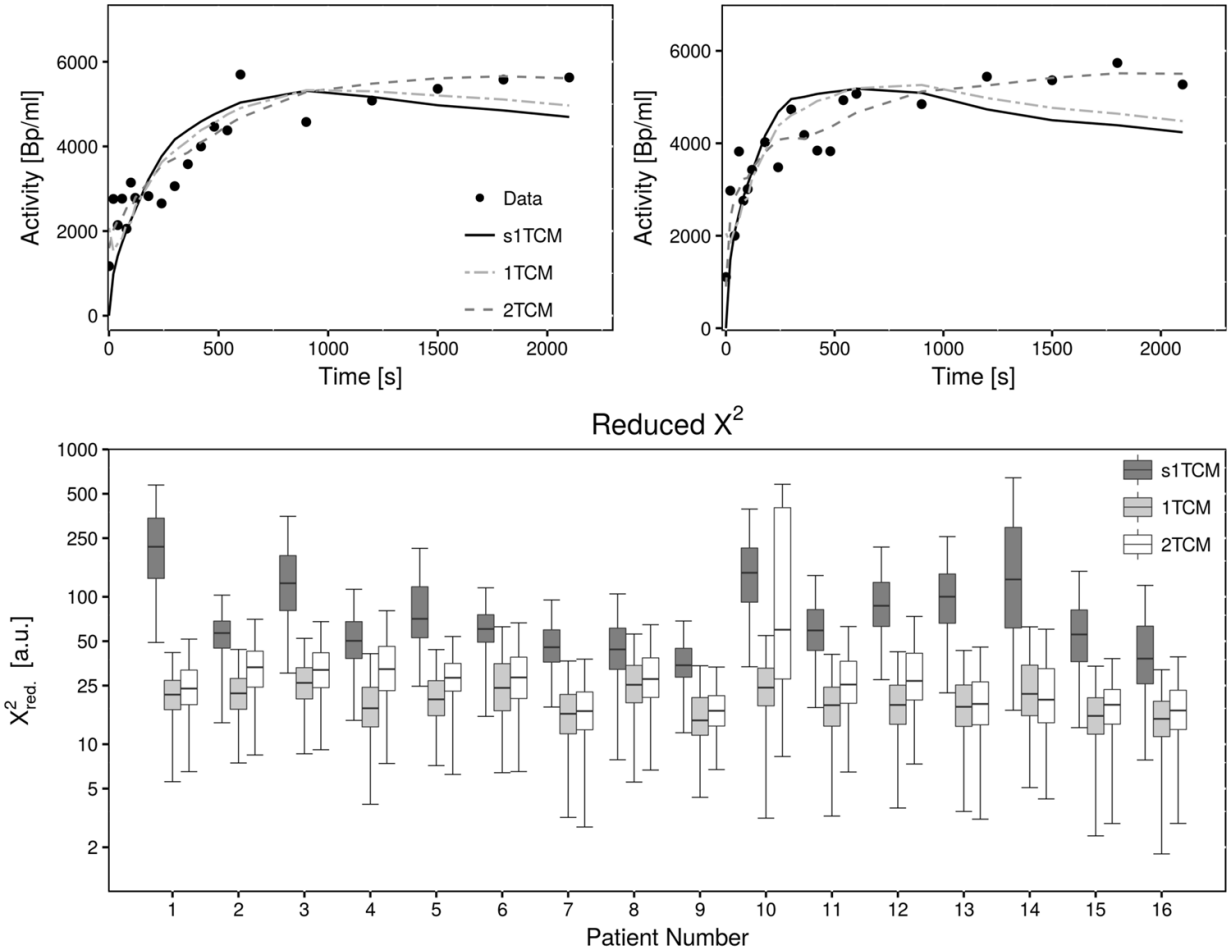
Goodness-of-fit in patient data from fits with different compartment models. The top two plots show the measured time-activity-curve in two exemplary voxels (dots) together with fitted curves from the 1TCM, standard 1TCM and 2TCM (lines). The 2TCM curve visually best describes the measured data points. Differences between s1TCM and 1TCM appear to be small. The bottom plot shows the distribution of the fit-quality measure reduced X² from all three models in every patient. Note that values for ${{\chi }^{2}}_{red}$ are shown on a logarithmic scale, since s1TCM yields much higher ${{\chi }^{2}}_{red}\,$than 1TCM and 2TCM. Lower values of ${{\chi }^{2}}_{red}$ indicate better fits, i.e. less deviation between model curve and data points. Fits from s1TCM yield significantly higher ${{\chi }^{2}}_{red}\,$, thus poorest fits. ${{\chi }^{2}}_{red}$ from the 1TCM and 2TCM yield similar values, however 1TCM appears to present with slightly better fits.

Quality of respective fits with the three different models was investigated. Figure 3 shows the measured TAC in two exemplary voxels together with fitted curves from all three models. It can be observed that the 2TCM model function fits the measured time-activity-curves best. The simplified 1TCM provides the least adequate fit, with substantial deviations from the measured curve, especially at later time points. The standard 1TCM describes the time course of tracer activity more appropriately; however, it also shows deviations towards the end of the acquisition. It could be hypothesized that the 2TCM describes the tracer kinetics most accurately, as it visually gives the best fit to the data. In order to further investigate this finding, quantitative analysis of goodness-of-fit was performed by evaluation of ${{\chi }^{2}}_{red}\,$and cAIC.

Reduced ${{\chi }^{2}}_{red}\,$from fits to data of each patient with either one of the three utilized models is shown in Fig. 3. The simplified 1TCM yielded much higher ${{\chi }^{2}}_{red}\,$compared to the standard 1TCM and 2TCM, indicating that it gives only a poor description of the TACs and thus tracer kinetics. ${{\chi }^{2}}_{red}$ from the standard 1TCM and 2TCM proved to be very similar. However, in most cases the 1TCM yielded slightly lower ${{\chi }^{2}}_{red}\,$values. On the other hand it showed more outliers to extremely high values in many patients, showing that in some voxels the model curve did not fit the data at all.

Regardless of the model, ${{\chi }^{2}}_{red}\,$takes very large values in all patients, even though it should be close to one. A possible explanation for this deviation is an underestimation of the acquisition noise.

**Figure 4:**
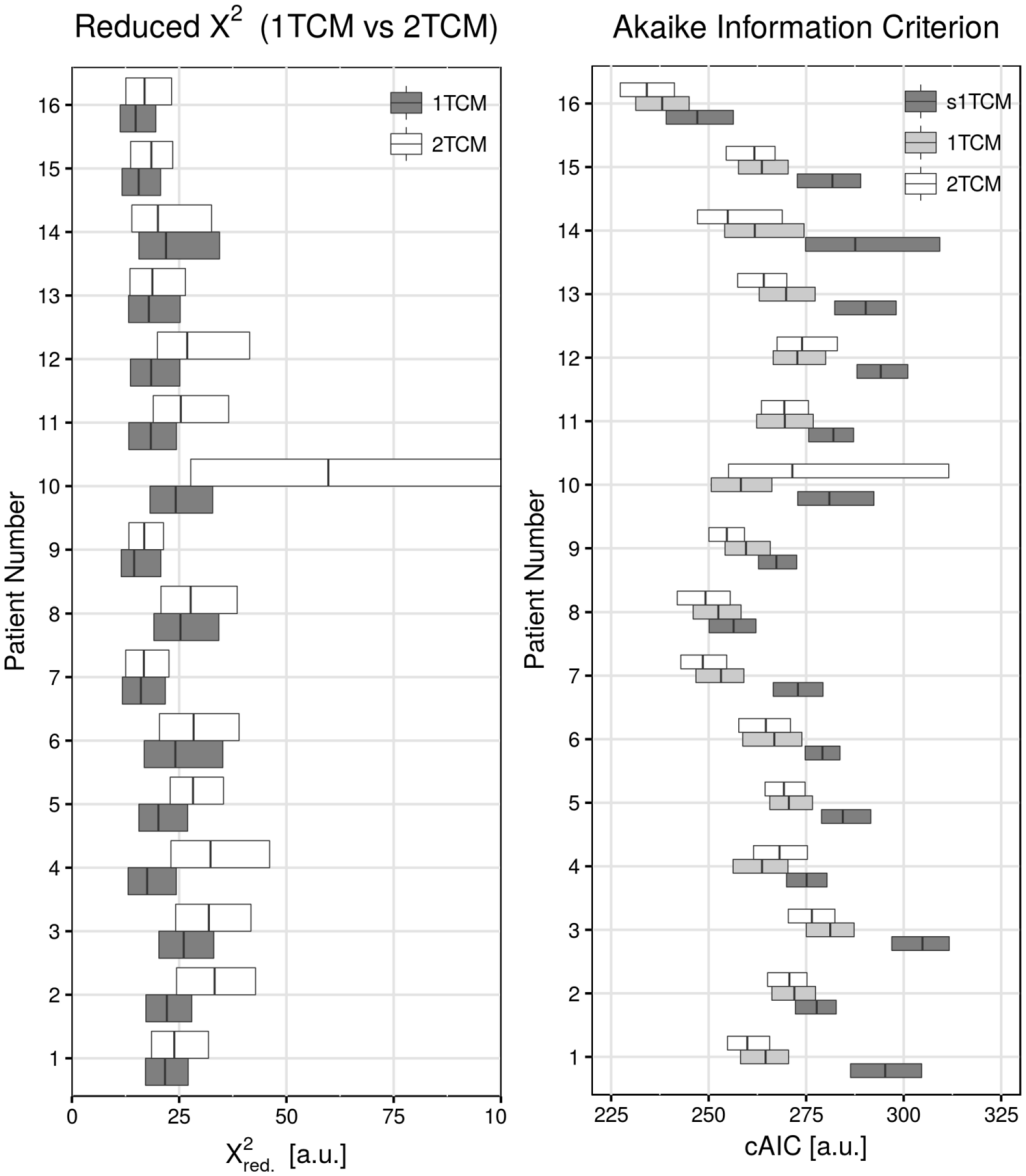
Goodness of fit compared to model stability. The left plot shows ${{\chi }^{2}}_{red}$ for the 1TCM and 2TCM on a zoomed scale for better comparison. The right plot shows the values for the corrected Akaike information criterion (cAIC) that accounts for model complexity by panelizing the fit quality (sum of squared residuals) with the number of parameters in the model. Lower cAIC indicates a better representation of the fitted data by the model. cAIC from the 2TCM were lowest, even though ${{\chi }^{2}}_{red}$ of this model was slightly higher compared to the 1TCM.

In order to consider fit quality with respect to the number of model parameters, the corrected AIC was calculated (Fig. 4). The results show that the simplified 1TCM yields the poorest results, however, fit quality according to the cAIC is not as bad as suggested by ${{\chi }^{2}}_{red}$. Interestingly, the 2TCM appears to be the best representation of the data (lowest cAIC) in most cases, even though fits showed slightly higher ${{\chi }^{2}}_{red}\,$compared to the 1TCM.

### Parameter estimates from patient data

**Figure 5:**
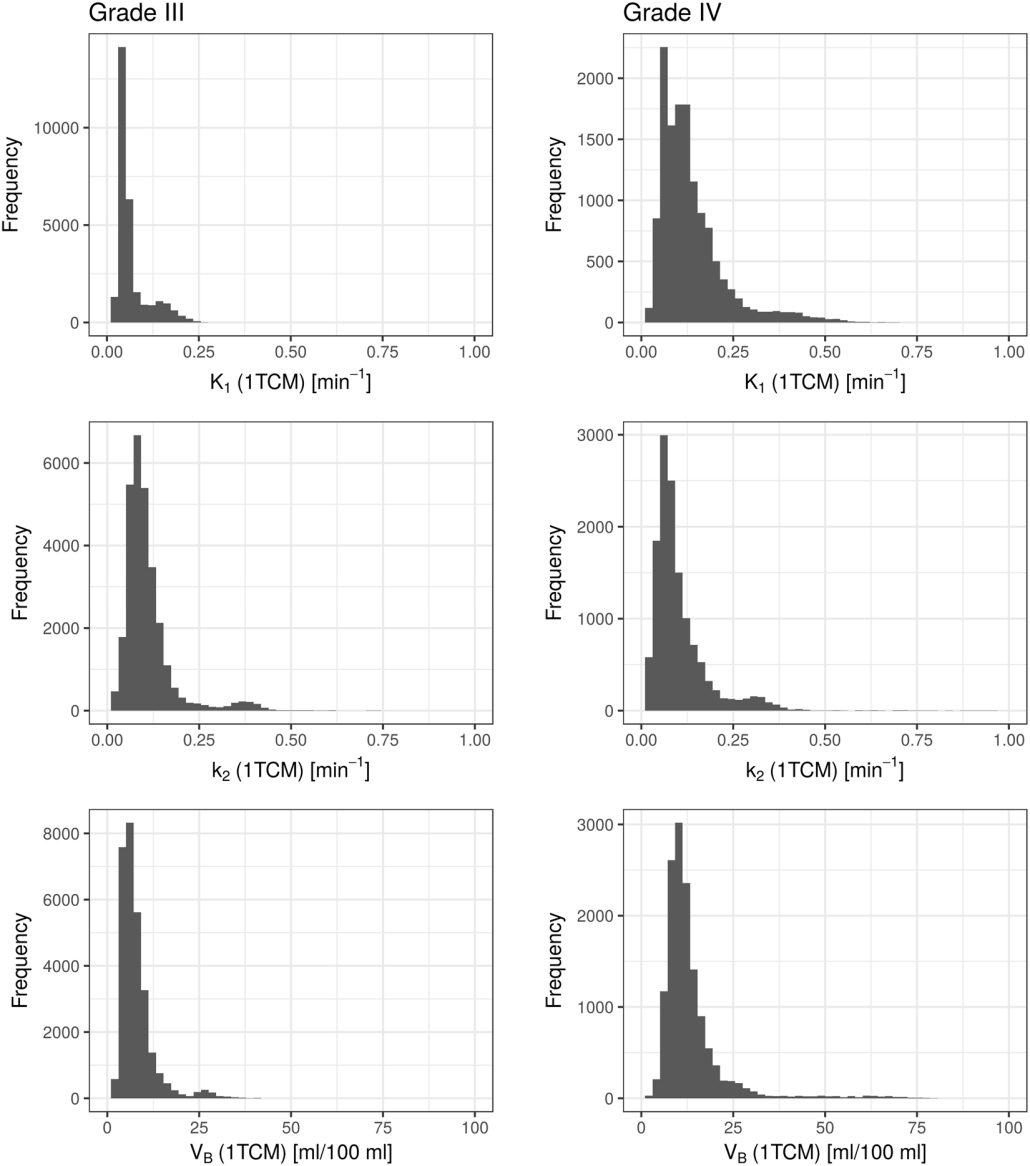
Histogram of total voxel frequency of parameter estimates on K_1_, k_2_ and V_B_ (from top to bottom) from fits with the 1TCM in grade III tumors (left column) and grade IV tumors (right column), respectively. A clear shift towards higher values for grade IV tumors can be seen for K_1_, and to some extend for V_B_, whilst distributions for k_2_ are relatively similar.

**Figure 6:**
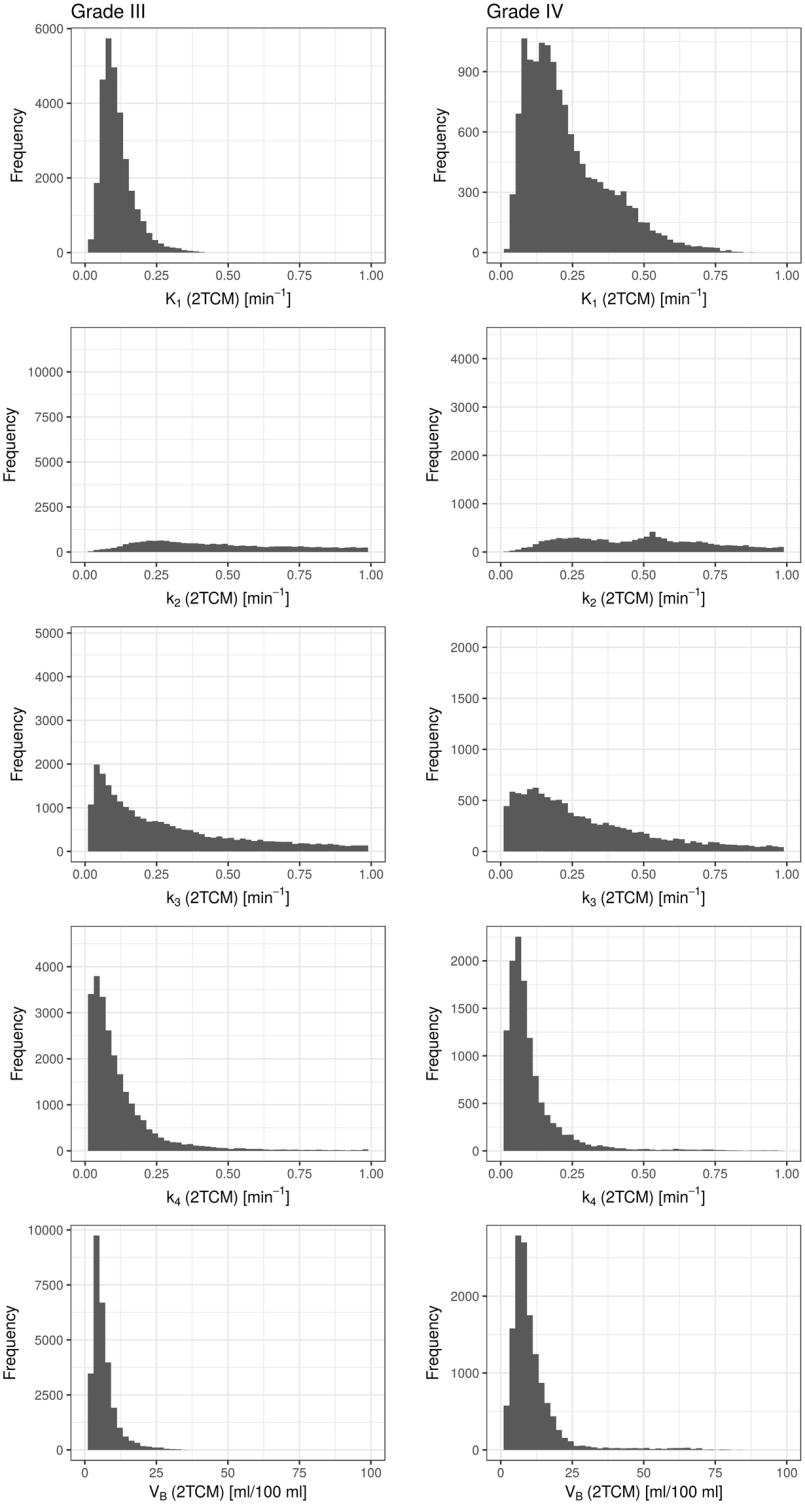
Histogram of total voxel frequency of parameter estimates on K_1_, k_2_, k_3_, k_4_ and V_B_ (from top to bottom) from fits with the 2TCM in grade III tumors (left column) and grade IV tumors (right column), respectively. A clear shift towards higher values for grade IV tumors can be seen for K_1_ and k_2_, and to some extend for V_B_ and k_3_. Distributions for k_4_ are relatively similar.

Table 2 lists median parameter estimates from the three models for all patients, together with the respective SUV_max_ and SUR values. Except for k_2_ from the 2TCM, which takes unreasonably high values in many patients (up to the constraint boundary of 1 min^−1^), parameter estimates from fits with all models present with reasonable values. Figures 5 and 6 show histograms of the total voxel frequency of parameter estimates from the 1TCM (Fig. 5) and 2TCM (Fig. 6) in patients with grade III and grade IV tumors, respectively.

**Table 2:** Median parameter estimates from fits with the three different compartment models in every patient, together with SUV_max_ and SUV-to-background ratio (SUR): K_1_ and k_2_ from the simplified 1TCM; K_1_, k_2_ and V_B_ from the standard 1TCM; K_1_, k_2_, k_3_, k_4_ and V_B_ from the 2TCM.

Grade	SUV_max_	SUR	s1TCM		1TCM			2TCM				
			K_1_[10^−2^ min^−1^]	k_2_[10^−2^ min^−1^]	K_1_[10^−2^ min^−1^]	k_2_[10^−2^ min^−1^]	V_B_[ml/100 ml]	K_1_[10^−2^ min^−1^]	k_2_[10^−2^ min^−1^]	k_3_[10^−2^ min^−1^]	k_4_[10^−2^ min^−1^]	V_B_ [ml/100 ml]
III	1.7	2.1	6.6	10.4	4.9	6.8	4.2	9.3	59.2	18.4	4.1	2.2
	3.9	3.4	12.4	9.2	10.3	6.8	6.7	13.7	33.1	23.0	7.0	5.1
	1.9	1.6	7.3	11.8	4.5	6.2	7.9	7.4	34.4	15.2	3.7	6.6
	1.1	1.2	15.3	39.2	4.3	7.3	8.6	7.2	45.8	28.8	7.7	7.3
	3.3	2.7	18.9	15.1	14.7	10.7	9.3	22.2	47.2	19.0	8.7	5.9
	3.0	3.1	27.2	26.5	16.4	13.4	10.3	19.4	32.8	28.3	17.2	9.4
	1.2	1.4	8.9	20.5	4.6	9.3	5.7	9.6	100	30.6	6.7	4.8
IV	1.9	1.7	23.9	43.3	7.2	8.7	12.6	10.1	51.8	35.1	7.5	11.4
	2.9	3.7	42.7	33.8	33.3	22.5	14.7	47.9	100	22.1	13.7	11.5
	4.6	3.2	23.2	15.6	14.8	7.5	15.9	21.8	49.8	44.8	8.4	13.4
	3.3	2.9	22.6	18.0	18.5	13.5	9.8	38.7	100	17.5	6.7	6.9
	3.4	4.1	17.1	15.3	12.6	9.6	11.5	20.4	46.7	18.5	7.4	8.8
	2.9	3.0	11.7	7.5	9.0	4.5	11.2	17.5	63.4	26.3	2.2	7.2
	3.4	3.4	13.9	11.0	9.9	6.5	8.7	20.0	97.9	30.6	5.9	5.8
	1.9	2.6	12.7	17.5	5.8	5.0	9.4	8.5	56.2	56.3	6.6	9.1
	2.8	2.6	14.7	14.7	10.4	7.4	10.4	16.2	42.3	23.8	7.5	7.7

Note: Median Parameter estimates were used instead of averages, due to several voxels with failed fits, which yielded outliers towards high transfer rates (~100 10^−2^ min^−1^ was set as upper constraint for all parameters).

**Figure 7:**
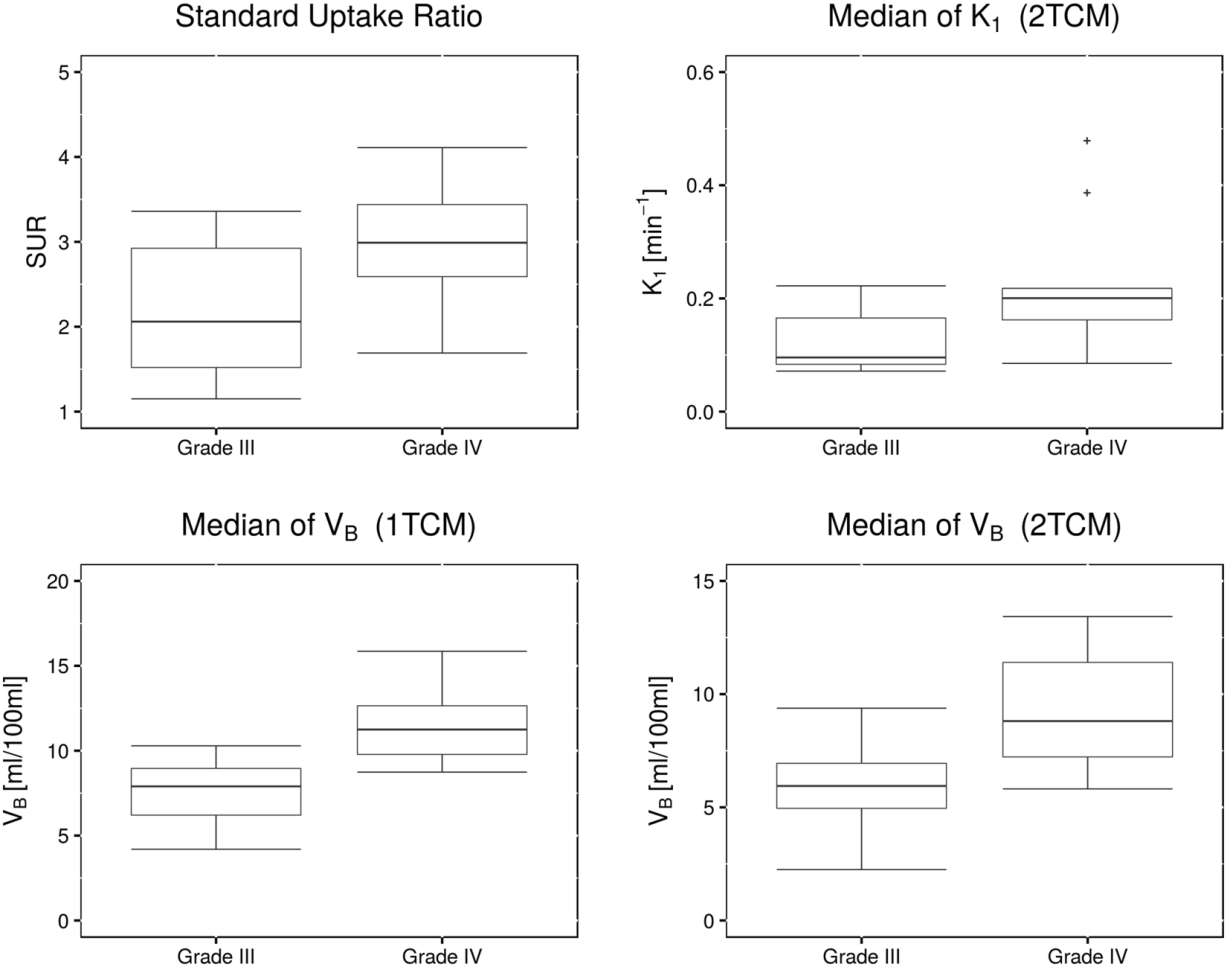
Correlation of parameter estimates with patient’s tumor grade. The plots display the differences in median parameter estimates between patients with WHO grade III and WHO grade IV gliomas. Mean values over the entire cohort were 2.2 vs. 3.0 for SUR (*p* = 0.09073), 7.5 ml/100 ml vs. 11.6 ml/100 ml for V_B_ from 1TCM (*p* = 0.0021), 0.127 min^−1^ vs. 0.224 min^−1^ for K_1_ from 2TCM (*p* = 0.09073) and 5.9 ml/100 ml vs. 9.1 ml/100 ml for V_B_ from 2TCM (*p* = 0.03112).

On average, patients with grade IV glioma presented with higher SUV_max_ and SUR (Fig. 7). The difference was not significant (SUV_max_: 2.3 vs. 3.0, *p* = 0.2; SUR: 2.2 vs 3.0, *p* = 0.091). K_1_ from fits with the simplified 1TCM showed a tendency towards higher values in patients with grade IV tumors, with mean values of K_1_ = 0.138 min^−1^ in grade III, and K_1_ = 0.204 min^−1^ in grade IV (*p* = 0.17). k_2_ from s1TCM fits was similar in both groups (k_2_ = 0.189 min^−1^ in grade III and k_2_ = 0.194 min^−1^ in grade IV). As already discussed, the standard 1TCM yielded lower values for K_1_ and k_2_ compared to the simplified 1TCM. However, the tendency of lower K_1_ in grade III tumors and k_2_ being alike in both entities remained (K_1_ = 0.085 min^−1^ vs. 0.135 min^−1^; k_2_ = 0.087 min^−1^ vs. 0.095 min^−1^). This can also be observed in the respective histogram, which presents with a clear shift towards higher values in grade IV tumors. V_B_ from the 1TCM fits was significantly lower in grade III glioma as shown in Fig. 7, with values of 7.5 ml/100 ml vs. 11.6 ml/100 ml (*p* = 0.02518, Bonferroni-Holmes corrected). This effect is also partially visible in the total voxel-value frequency histogram. For the 2TCM, similar results were observed with V_B_ (grade III) = 5.9 ml/100 ml vs. V_B_ (grade IV) = 9.1 ml/100 ml (*p* = 0.03112). The result did not remain significant after multiple testing corrections.

Average K_1_ in the 2TCM over all patients yielded significantly lower values in grade III compared to grade IV glioma (0.127 min^−1^ vs. 0.224 min^−1^, *p* = 0.0091, Fig. 6 top). Estimates on k_2_ presented with a tendency to unrealistically high values in both groups (see Table 2): k_2_ (grade III) = 0.503 min^−1^ and k_2_ (grade IV) = 0.676 min^−1^. Furthermore, estimates on k_2_ resulted in several outliers up to the upper parameter constraint of 1 min^−1^ especially in grade IV tumors. Thus, it is likely, that the fits in these voxels ran into constraint boundaries. Values for k_3_ showed a trend to higher values in grade IV tumors (0.233 min^−1^ vs. 0.305 min^−1^), whereas mean k_4_ was comparable with 0.079 min^−1^ in grade III and 0.073 min^−1^ in grade IV.

Parameter estimates from the different models, as well as grade and SUR, were further correlated with patient’s overall survival (OS) and progression free survival (PFS). Results are listed in Supplementary Table S1. SUR showed correlation with OS (HR = 2.78, CI = [1.17; 6.64], *p* = 0.021). Furthermore, median K_1_ from both the 1TCM and 2TCM fits showed significant correlation with OS: HR = 2.78, CI = [0.99; 7.84], *p* = 0.043 (1TCM); HR = 3.02, CI = [1.07; 8.59], *p* = 0.036 (2TCM). For both models, a cut-off for K_1_ was found, that significantly separated patients overall survival, as shown in Supplementary Fig. S2. Patients with lower K_1_ showed improved survival, with thresholds of K_1_(1TCM) = 0.074 min^−1^ (*p* = 0.0041) and K_1_(2TCM) = 0.104 min^−1^ (*p* = 0.0041), respectively. Cut-offs for K_1_ from both models also presented with tendencies to separate progression free survival: K_1_(1TCM) = 0.059 min^−1^, *p* = 0.082 and K_1_(2TCM) = 0.104 min^−1^, *p* = 0.086. A trend towards correlations of these parameters with PFS was found, that was however not significant (*p* = 0.2006 and *p* = 0.154, respectively). Furthermore, k_2_ from the 1TCM showed tendencies towards correlation with PFS (p = 0.1042). Parameter k_4_ separated progression free survival with a threshold of k_4_ = 0.067 min^−1^ (*p* = 0.066). Cox proportional hazards model revealed however non-significant correlations with *p* = 0.301.

### Evaluation of accuracy and robustness of the 2TCM on simulation data

**Figure 8:**
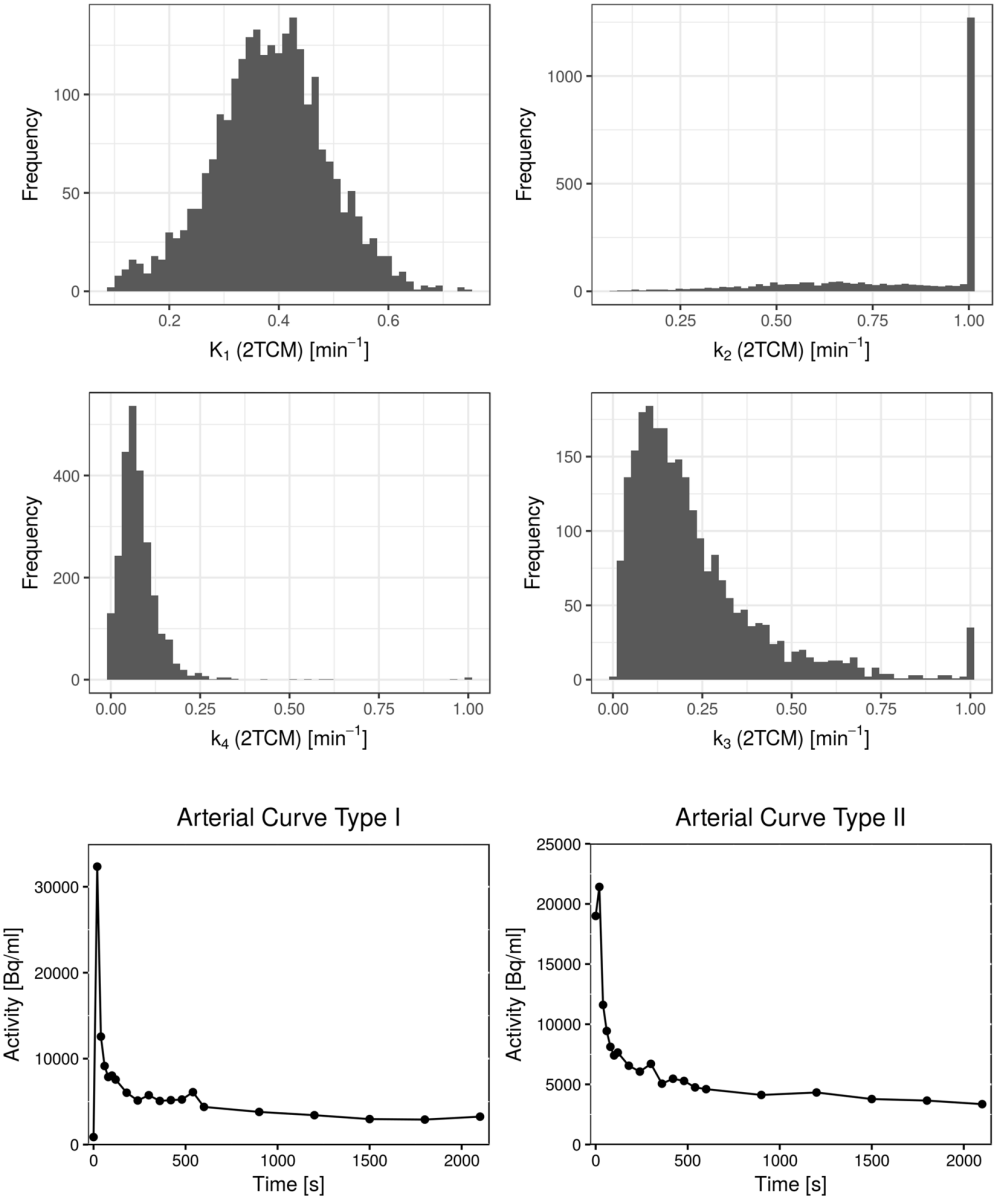
Extraction of parameter values for simulation time-activity curves with the 2TCM. The top plots show representative histograms of the distributions of parameter estimates K_1_, k_2_, k_3_ and k_4_ from fits with the 2TCM in an exemplary patient. Peak analysis of the histograms of all patients was used to identify the most common value of these parameters that were used as input for data simulation. Two different AIFs, displayed in the bottom two plots, were used for both data simulation and subsequent fitting.

In order to investigate the validity of 2TCM and test accuracy and robustness of the determined parameter estimates, histogram analysis of the individual patient’s parameter maps was performed, as illustrated in Fig. 8 for one patient. Corresponding histograms for K_1_ and k_2_ from the 1TCM are shown in the supplements (Fig. S3). Peak values of the histograms of each parameter were extracted in order to find common parameter values [K_1_, k_2_, k_3_, k_4_, V_B_]. From these values, 10 combinations were composed for data simulation, which are listed in Table 3. These parameter combinations together with two measured AIFs (Fig. 8 bottom) were used to simulated time-activity curves, which were fitted again with the 2TCM. The resulting parameter estimates were evaluated by means of stability and precision.

**Table 3:** Parameter values for K_1_, k_2_, k_3_, k_4_ and V_B_ from the 2TCM used for simulation of time activity curves.

Combination	K_1_[10^−2^ min^−1^]	k_2_[10^−2^ min^−1^]	k_3_[10^−2^ min^−1^]	k_4_[10^−2^ min^−1^]	V_B_ [ml/100 ml]
1	10	20	2.5	5	5
2	10	20	7.5	5	5
3	10	60	12.5	5	5
4	10	45	7.5	5	5
5	10	20	12.5	5	5
6	20	20	2.5	5	5
7	20	20	7.5	10	5
8	20	60	12.5	5	5
9	40	45	2.5	20	5
10	40	60	7.5	5	5

Each parameter combination was used with either one of the two AIF types (Fig. 6) to simulated 10000 curves. Gaussian random numbers were added to the simulated curves, in order to simulate noise at the same level as the measured patient TACs. These simulated data were in terms fitted with the 2TCM in order to evaluate precision and robustness of parameter estimates from the 2TCM in data of our quality (temporal resolution, sampling, SNR).

**Figure 9:**
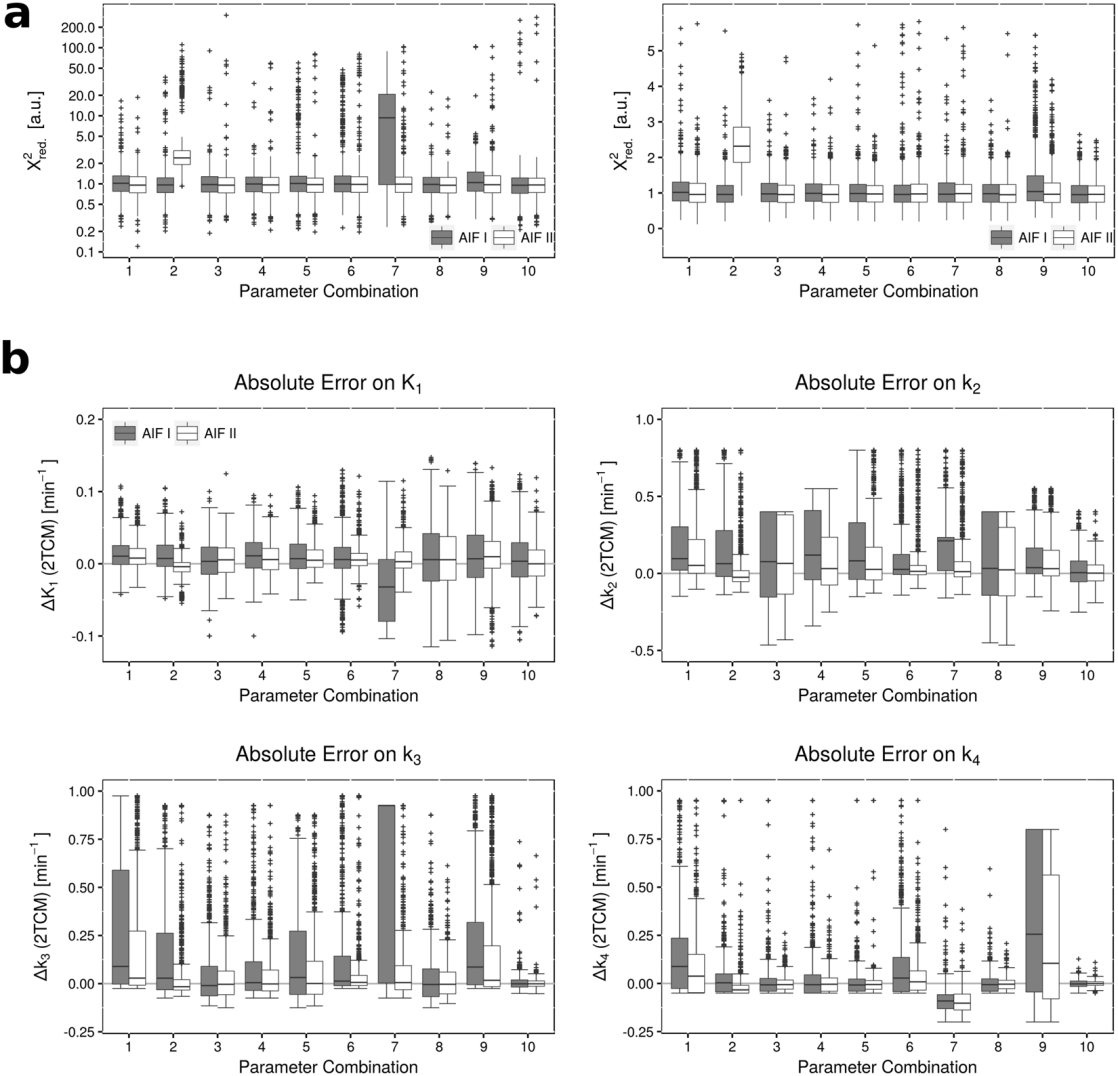
Results from fitting simulated data with the 2TCM. Figure 7 (**a**) shows goodness-of-fits in terms of reduced X², for each of the 10 different parameter combinations (with both AIFs). Due to outliers at very high values, the y-axis in the left plot is displayed on a logarithmic scale. For more detailed evaluation, the right plot shows the distribution of X² at an enlarged linear scale. Apart from two exceptions, both AIF types yielded similar fit quality. Figure 7 (**b**) shows the distribution of absolute errors in each of the four model parameters K_1_, k_2_, k_3_ and k_4_. The absolute error was calculated as the parameter estimate minus the true value used for simulation. While K_1_ yields good accuracy and robustness, k_2_ showed lowest stability of all parameters. k_3_ and k_4_ showed tendencies towards positive values for the errors, indicating a systematic overestimation of these two parameters.

Figure 9a shows the distribution of ${{\chi }^{2}}_{red}$ for all 10 combinations, with either one of the AIFs. Fits to data simulated with combination 7 and AIF type I, as well as combination 2 with AIF type II, yielded exceptionally poor fit quality. Apart from these cases, fit quality was good, with ${{\chi }^{2}}_{red}\,$being close to one. However, in all combinations, some fits completely failed, yielding outliers of very high ${{\chi }^{2}}_{red}$. This suggests decreased stability of the fits, which could be overcome by averaging signal from multiple voxels and thus, decreasing noise of the TACs (ROI-based analysis).

Figure 9b shows absolute errors on parameter estimates of K_1_, k_2_, k_3_ and k_4_. It can be observed, that fits with AIF type II yield overall more stable results, with smaller quantiles of the parameter value distribution. This is counter-intuitive, since AIF type I would be assumed to yield better fit accuracy, because it captures the peak activity better. Interestingly, the distinct difference between the two AIF types was not observed in fit quality.

Overall, both accuracy and stability varied greatly over the different parameter combinations, which suggests that the 2TCM accurately describes the kinetics only in some tissue settings. Estimates on K_1_ showed the lowest errors of all, with deviations of about 0.01 min^−1^. k_2_ presents with increased errors, which are nonetheless not larger than 0.5 min^−1^ within the 25^th^ and 75^th^ quantile. With regards to stability, k_2_ showed the widest distribution of parameters, suggesting that it is the least stable parameter. k_3_ shows large errors for combinations 1 and 7 (AIF type I). The errors in combination 7 were already suggested by the poor fit quality and high ${{\chi }^{2}}_{red}$. Interestingly, combination 2 (AIF type II) showed low errors on parameter estimates, even though ${{\chi }^{2}}_{red}\,$predicted the fits to be of poor quality. In all combination k_3_ shows many outliers to high values, indicating failed fits. k_4_ shows good stability and accuracy, except for one case (combination 9). This combination is the only one with a large true value of k_4_ = 0.2 min^−1^, showing that the model loses robustness for high values of this parameter. The inability of the 2TCM do deal with high values of k_4_ could also be an explanation for the poorer fit quality in combination 7, where k_4_ was set to 0.1 min^−1^.

In the majority of cases k_3_ and k_4_ show positive errors, meaning that they are commonly overestimated by the fit. This is partly due to the fact that they were initial set to very low values for simulation, which also makes it difficult to interpret accuracy of these parameters in terms of absolute errors. Thus, further analysis was done by evaluation of relative errors on parameter estimates, which are displayed in Table 4 for the different parameter and AIF combinations. The high stability and accuracy of estimates on K_1_ is confirmed, as the parameter shows low errors, which are mostly under 10% and quantile errors do not exceed ± 30%. k_2_ shows errors that are increased in several cases up to ~50%, but mostly low as well. However, in 4 cases, the deviations from the true value are substantial, because the quantiles do not include the 0% error value. k_3_ and k_4_ show large deviations from the true values in combinations 1, 7 and 9, with median errors up to several hundred percent with AIF type I. Furthermore, even though median errors are low in most of the other combinations, the parameters present with increased instability, reflected in the large quantiles of the errors.

**Table 4:** Median relative errors on parameter estimates from fitting simulated data of 10 different parameter combinations (Table 3) and two different AIFs with the 2TCM, for each of the model parameters K_1_, k_2_, k_3_ and k_4_ and V_B_.

Combination	AIF	ΔK_1_ [%]	Δk_2_ [%]	Δk_3_ [%]	Δk_4_ [%]	ΔV_B_ [%]
1	I	10.5	47.9	356.9	176.0	−7.0
		[−1; 25]	[10; 151]	[−9; 2357]	[−52; 471]	[−23; 11]
	II	7.9	25.9	115.6	76.0	−5.3
		[−1; 21]	[1; 110]	[−34; 1091]	[−95; 302]	[−16; 6]
2	I	7.4	31.5	38.0	6.8	−6.6
		[−4; 26]	[−10; 139]	[−43; 349]	[−87; 100]	[−24; 11]
	II	−4.0	−12.5	−21.1	−66.9	−8.8
		[−11; 2]	[−26; 9]	[−44; 28]	[−100; −19]	[−19; 5]
3	I	3.3	12.7	−8.1	−14.5	−0.5
		[−15; 23]	[−26; 67]	[−51; 72]	[−81; 53]	[−18; 17]
	II	5.4	10.8	−2.6	−12.1	−1.5
		[−12; 22]	[−22; 63]	[−46; 52]	[−59; 36]	[−13; 13]
4	I	11.0	26.4	6.8	−12.8	−4.1
		[−6; 29]	[−9; 91]	[−47; 152]	[−100; 90]	[−21; 14]
	II	5.8	6.9	−2.0	−7.9	−1.9
		[−8; 21]	[−17; 52]	[−50; 94]	[−81; 57]	[−16; 10]
5	I	7.1	40.9	25.5	−16.0	−3.4
		[−6; 27]	[−19; 165]	[−45; 218]	[−81; 46]	[−21; 16]
	II	5.0	12.9	0.5	−10.8	−5.3
		[−5; 19]	[−21; 85]	[−44; 93]	[−60; 30]	[−18; 8]
6	I	2.9	13.0	52.3	56.4	−1.3
		[−3; 11]	[−4; 62]	[−43; 571]	[−85; 271]	[−19; 18]
	II	2.6	6.7	25.8	16.8	−2.4
		[−1; 7]	[−4; 26]	[−40; 171]	[−69; 131]	[−15; 11]
7	I	−16.1	105.7	1233.3	−45.5	191.6
		[−40; 3]	[9; 117]	[−4; 1233]	[−66; −29]	[−4: 282]
	II	1.4	5.7	7.4	−51.1	−0.4
		[−3; 8]	[−11; 38]	[−43; 125]	[−69; −28]	[−13; 12]
8	I	2.8	5.4	−3.3	−12.0	799.2
		[−12; 21]	[−24; 67]	[−54; 61]	[−82; 48]	[780; 815]
	II	2.8	3.8	−2.6	−7.3	798.5
		[−11; 19]	[−24; 50]	[−43; 48]	[−54; 33]	[786; 811]
9	I	1.7	8.3	344.8	127.8	3.1
		[−5; 10]	[0; 37]	[−23; 1272]	[−22; 400]	[−19; 33]
	II	2.5	6.8	69.5	52.4	−2.0
		[−2; 8]	[−4; 33]	[−63; 786]	[−40; 281]	[−16; 14]
10	I	0.9	0.8	−1.1	−4.7	−1.4
		[−5; 7]	[−9; 13]	[−24; 26]	[−31; 25]	[−20; 17]
	II	0.0	0.3	−1.1	−1.0	−0.1
		[−4; 5]	[−8; 9]	[−18; 19]	[−20; 18]	[−13; 12]

The 25^th^ and 75^th^ quantile of the errors are denoted in square brackets under the median value. Normalizing the absolute error to the true parameter value used for simulation, and multiplying by 100 calculated relative errors. Quantiles of k_2_ show, that even though errors on this parameter are low in most cases, it presents with high instability. k_3_ and k_4_ show large relative errors, which is likely to be due to their low original values.

## Discussion

The presented study investigated the feasibility of tracer kinetic analysis in dynamic ^18^F-FET PET scans of patients with recurrent high-grade glioma re-irradiated with carbon ions. Up-to-date, controversial reports exits on the value of SUV and SUV-to-background ratio (SUR) for grading of glioma^17,21,31,33^. These discrepancies might be linked to differences in SUR depending on the time point of PET image acquisition after injection of the tracer, as demonstrated by Weckesser *et al*.^18^. In this context, dynamic ^18^F-FET PET may provide more detailed information about tracer uptake compared to static image acquisition. However, dynamic scanning protocols are rarely used in clinical routine, due to the time-consuming acquisition and challenging interpretation of the data.

There are only few reports on detailed compartment model analysis in ^18^F-FET PET in patients, and the question, which model best describes the kinetics of tracer uptake, metabolism and washout is still unanswered^23,24^. However, voxelwise tracer kinetic analysis holds the promise of unraveling tumor sub-volumes of different metabolic activity, which is especially relevant in high-grade glioma due to their infiltrative and heterogeneous nature. Compartment modeling can yield direct physiological correlates on the blood volume and perfusion (i.e. vascularization) and metabolic rate constants.

Retrospective data of 16 patients were analyzed by voxelwise fitting of measured time-activity curves (TAC) with three different compartment models: a standard one-tissue compartment model (1TCM), a simplified 1TCM that assumes the blood volume to be zero (s1TCM) and a two-tissue compartment model (2TCM).

All models could be fitted to the data appropriately within an isocontour of 70%. The applied acquisition protocol was feasible for tracer kinetic analysis with compartment models, providing sufficient data quality in terms of temporal resolution and signal-to-noise ratio. We could find no evidence against application of either of the models on clinical patient data. The standard 1TCM and 2TCM fitted the data very well, with good fit quality and reasonably low ${{\chi }^{2}}_{red}$, however, the 1TCM yielded failed fits with exceptionally high ${{\chi }^{2}}_{red}$ in several cases. The simplified 1TCM with V_B_ = 0 presented with poorer fits and the model did not properly represent the curve shapes. Both versions of the 1TCM yielded reasonable estimates on transfer rates K_1_ and k_2_. The 2TCM yielded the least stable parameter estimates, especially for k_2_. There were no hints towards overfitting of the data with the 2TCM, as in fact it yielded higher ${{\chi }^{2}}_{red}$ (lower fit quality) than the standard 1TCM.

Estimates on the blood volume V_B_ were reasonable and showed similar patterns of increased and decreased values for the 1TCM and 2TCM, indicating that this parameter is not negligible and the simplified 1TCM might not be an adequate description of uptake kinetics. This is conceivable, as high-grade glioma present with enhanced vascularization and thus, a large blood volume, whereas the fraction of blood vessels is approximately 2 to 4% in normal brain tissue^25^.

A large source of error in tracer kinetic modeling is the selection of an appropriate arterial input curve^34^. Ideally, the AIF would be derived from blood sampling during tracer administration; however, this invasive procedure is rarely feasible with patients. Furthermore, in retrospective settings, such an AIF is impossible to acquire. Therefore, image-derived input functions are often chosen, even though they are prone to error due to partial volume effects, motion during acquisition, etc. In this study, image-derived input functions were carefully extracted from carotid arteries, applying several suggested methods^34^ like co-registration with MRI and visual inspection of TACs in included voxels, in order to minimize distortion of the arterial signal. Nonetheless, no ground truth AIF was available for comparison, and underestimation of arterial tracer concentration, especially in the peak region, can influence parameter estimates of kinetic modeling. No relation was found between the quality of fits and the AIF type, even though it was assumed that fits with AIFs of type III could yield poorer results due to an underestimation of the peak activity.

In many cases, temporal resolution is increased for the initial frames in order to more precisely sample the peak concentration of the AIF and hence, improve parameter estimates. In principle this is possible, as most PET dynamic data is acquired in list mode and afterwards sampled to respective time frames at reconstruction. However, higher temporal resolution always comes at the cost of reduced SNR, which on the other hand decreases quality of the extracted arterial TAC and thus, fit quality^35^. In contrast to MRI, the signal from the reconstructed PET images is the accumulation of all decays measured between time frames, thus forming an integral signal. This aspect potentially reduces the risk of underestimating the peak activity in the AIF.

It was found that the parameter V_B_ provides a diagnostic value for tumor grading, especially in the standard 1TCM. Overall V_B_ from the 1TCM was 7.5 ml/100 ml for grade III tumors and 11.6 ml/100 ml for grade IV tumors (*p* = 0.02518 after multiple-testing correction). Using the 2TCM yielded lower estimates for V_B_ of 5.9 ml/100 ml in grade III glioma and 9.1 ml/100 ml in grade IV glioma. These estimates are comparable to values published in the literature, that were derived using dynamic contrast-enhanced MRI (DCE MRI)^36,37^. Prediction of tumor grade in recurrent glioma via imaging has a clinical impact, as these tumors may derive after long-latency period from lower-grade glioma and are often no more biopsied. Therefore, differentiation of tumor grades based on dynamic ^18^F-FET PET scans could provide valuable input in predicting the outcome in recurrent setting.

Parameter estimates were further correlated with tumor grade and OS. Parameter estimates from the 2TCM hinted towards correlation with patients’ survival, further indicating the additional value of detailed tracer kinetic analysis in ^18^F-FET PET. Additional studies are required to validate potential correlations between 2TCM parameters and OS.

With the 2TCM being more detailed but also less stable compared to the 1TCM, the question arises, which model fit the data more adequately, and thus, better represents the underlying tracer kinetics. Therefore, accuracy and robustness of the more complex 2TCM were investigated by simulating TACs of 10 tissue types and fitting this synthetic data with the 2TCM. Since no complete set of reference values for exchange rates K_1_, k_2_, k_3_, k_4_ and V_B_ was given by the literature, we determined 10 common parameter combinations in our patient data and used these, together with measured patient AIFs, to simulate time-activity curves at the same data quality as the patient PET scans.

Fit quality and stability as well as errors on parameter estimates were quantified in order to assess reliability of this model for data at the same quality (SNR, temporal resolution) as patient scans. Results showed sufficient fit quality and precision of parameter estimates. K_1_ and V_B_ are parameters with high stability and accuracy. k_3_ and k_4_ exhibit large errors at large values (~0.20 min^−1^) but are otherwise robust estimates. k_2_ proved to be the least stable and least reliable parameter. Interestingly, AIFs of type II, where the initial frame already includes enhanced activity instead of the zero base line, provided better fit-quality and smaller errors, contrary to the expectations. It was initially hypothesized that a zero-activity first frame is required for a proper description of the peak activity.

Several authors have studied the value of dynamic ^18^F-FET PET for grading and prognosis in both low- and high-grade glioma^9,10,20,22,38^. Pöpperl *et al*. showed the superior value of dynamic acquisition protocols over static ones for grading in glioma patients^21^. However, for analysis of dynamic scans, most authors rely on qualitative description such as the overall tumor TAC shapes, the determination of semi-quantitative values like SUV at different time points^22^ or the time-to-peak activity^9,20^. To our knowledge this study provides the first quantitative report on detailed parameter estimates from these compartment models. Thiele *et al*., performed tracer kinetic modeling with the 1TCM and 2TCM amongst other analysis techniques of dynamic ^18^F-FET PET in grade IV glioma patients^23^. However, correlations of parameters from compartmental analysis with clinical outcome were missing due to the low stability of their results. Furthermore, the sinus sagittalis was used for derivation of image-based arterial TACs, which only gives the tracer concentration in venous blood. In addition, neither fit quality nor mentioned instability nor reliability of parameter estimates was further quantified. In contrast, the results of the current analysis provide an estimate on precision and robustness of tracer-kinetic analysis with the 2TCM by evaluating fits to simulated data.

Recently, pharmacokinetic modeling of ^18^F-FET tracer kinetics in a syngeneic orthotopic preclinical model was reported by injecting F98 glioblastoma cells into the right hemisphere of Fisher rats^39^. They found a favorable performance of the 2TCM in terms of model selection criteria AIC, *F* test and residual plots. This finding is in accordance with our study, showing that the 2TCM presents with lowest cAIC and thus appears to be the best representation of the data. However, there are some differences in their study compared to ours. First, F98 is carcinogen (nitrosourea-mediated mutagenesis) induced rat glioblastoma model that might differ not only with respect to the molecular characteristics affecting radiosensistivity (e.g., presence of BRCA1 mutation) but also tumor invasion and angiogenesis pattern from the human glioblastoma in patients^40,41^. A second pivotal difference between the two studies is that they do not consider the parameter blood volume (V_B_) in their compartment models. Indeed, we found that neglecting the blood volume in the 1TCM (simplified 1TCM) model yields poor results. However, high-grade glioma are highly vascularized necessitating inclusion of V_B_ in modeling these tumors, at least in patients. Together, our data underscore the importance of considering V_B_ in dynamic modeling. Moreover, we performed simulation analyses to investigate accuracy and robustness of parameter estimates. Results showed that the 2TCM is in principle feasible and accurate, however, at the cost of reduced fit stability.

Overall, the standard 1TCM provided the best results in terms of feasibility and robustness with an appropriate description of the tracer kinetics. This is in concordance with results reported in animal studies^24^. Additionally, correlation of K_1_ with overall patient survival was visible in K_1_ from both the 1TCM and 2TCM.

The blood volume V_B_ appears to be overestimated by the 1TCM compared to values reported previously in the literature^36^. Precision and robustness of the estimates from the 1TCM should be further investigated, e.g. with a simulation study similar to the one we conducted on the 2TCM in a larger population.

Fitting dynamic ^18^F-FET PET data with a 2TCM appears to hold some valuable information on tumor heterogeneity and substructure. The 2TCM provided the best model representation according to the Akaike information criterion^28,29^. Estimated V_B_ from the 2TCM yielded reliable values with good accuracy and robustness and showed a trend towards prognostic value for grading, as a trend towards higher values for K_1_ and V_B_ was observed in grade IV tumors compared to grade III tumors. k_4_ appears to correlate with progression free survival. Larger patient cohorts need to be analyzed to further investigate these findings. Furthermore, the combination of parameters from this more complex model with physiological measures derived from other imaging modalities (e.g. dynamic contrast-enhanced MRI) could provide additional, relevant information on tumor tissue composition.

## Conclusion

The presented study provides a first overview on the diagnostic and prognostic value of compartmental analysis for dynamic ^18^F-FET PET in recurrent high-grade glioma patients. The models revealed an improved resolution of tumor substructure (e.g. vascularization). Despite the limited size of the cohort, correlation of parameters with tumor grade and overall survival yielded promising results, which warrants further exploration. It is yet to be investigated, whether our findings can be translated to primary, untreated tumors. However, feasibility of the analysis is independent of this, enabling a proper conduction of such a study.

## Electronic supplementary material

Supplementary Figures
