# Supplementary material for: Feasibility and robustness of dynamic 18F-FET PET based tracer kinetic models applied to patients with recurrent high-grade glioma prior to carbon ion irradiation
Source: Sci Rep. 2018 Oct 3;8:14760. doi: 10.1038/s41598-018-33034-5 (PMC6170489; doi:10.1038/s41598-018-33034-5)
Supplement: Supplementary file 1 — Supplementary Figures [file 41598_2018_33034_MOESM1_ESM.docx]

Feasibility and robustness of dynamic ^18^F-FET PET based tracer kinetic models applied to patients with recurrent high-grade glioma prior to carbon ion irradiation

Charlotte Debus^1, 2, 3, 4, #^, Ali Afshar-Oromieh^5,6,7^, Ralf Floca^3,8^, Michael Ingrisch^9,6^, Maximilian Knoll^1, 2, 3, 4^, Jürgen Debus^1, 2, 3, 4^, Uwe Haberkorn^5*,^ and Amir Abdollahi^1, 2, 3, 4*^

*^1^German Cancer Consortium (DKTK), Heidelberg, Germany
^2^Translational Radiation Oncology, National Center for Tumor Diseases (NCT), German Cancer Research Center (DKFZ), Heidelberg, Germany
^3^Division of Molecular and Translational Radiation Oncology, Heidelberg University Medical School, Heidelberg Institute of Radiation Oncology (HIRO), National Center for Radiation Research in Oncology (NCRO), Germany
^4^Heidelberg Ion-Beam Therapy Center (HIT), Department of Radiation Oncology, Heidelberg University Hospital, Germany
^5^Department of Nuclear Medicine, Heidelberg University Hospital, Germany, ^6^Clinical Cooperation Unit Nuclear Medicine, German Cancer Research Center DKFZ), Heidelberg, Germany ^7^Department of Nuclear Medicine, Inselspital, Bern University Hospital, University of Bern, Switzerland
^8^Division of Medical Image Computing, German Cancer Research Center DKFZ), Heidelberg, Germany
^9^Department of Radiology, University Hospital Munich, Ludwig-Maximilians-University Munich, Germany*

** shared senior authors*

**Supplements**

|  | | OS | | |  | PFS | | |
| --- | --- | --- | --- | --- | --- | --- | --- | --- |
|  | | HR | CI | p |  | HR | CI | p |
| Grade | | 2.17 | [0.65; 7.29] | 0.211 |  | 1.44 | [0.42; 4.99] | 0.567 |
| SUR | | 2.78 | [1.17; 6.64] | 0.021 |  | 1.79 | [0.75; 4.3] | 0.191 |
| 1TCM | K_1_ | 2.78 | [0.99; 7.84] | 0.0429 |  | 1.98 | [0.68; 5.76] | 0.2006 |
|  | k_2_ | 0.84 | [0.18; 4] | 0.8255 |  | 3.83 | [ 0.74; 19.75] | 0.1042 |
|  | V_B_ | 1.74 | [0.38; 7.99] | 0.4632 |  | 3.52 | [0.49; 25.43] | 0.1738 |
| 2TCM | K_1_ | 3.02 | [1.07; 8.58] | 0.0356 |  | 2.14 | [0.75; 6.11] | 0.154 |
|  | k_2_ | 1.83 | [0.35; 9.55] | 0.4781 |  | 1.78 | [0.36; 8.78] | 0.478 |
|  | k_3_ | 0.78 | [0.16; 3.92] | 0.764 |  | 0.86 | [0.21; 3.56] | 0.831 |
|  | k_4_ | 1.08 | [0.27; 4.28] | 0.918 |  | 1.94 | [0.55; 6.81] | 0.301 |
|  | V_B_ | 1.70 | [0.49; 5.89] | 0.4 |  | 1.79 | [0.45; 7.09] | 0.411 |

**Supplementary Table S1:** Correlation of Grade, SUR, log transformed median parameter estimates from the 1TCM and 2TCM with overall survival (OS) and progression free survival (PFS), according to Cox proportional hazards model.


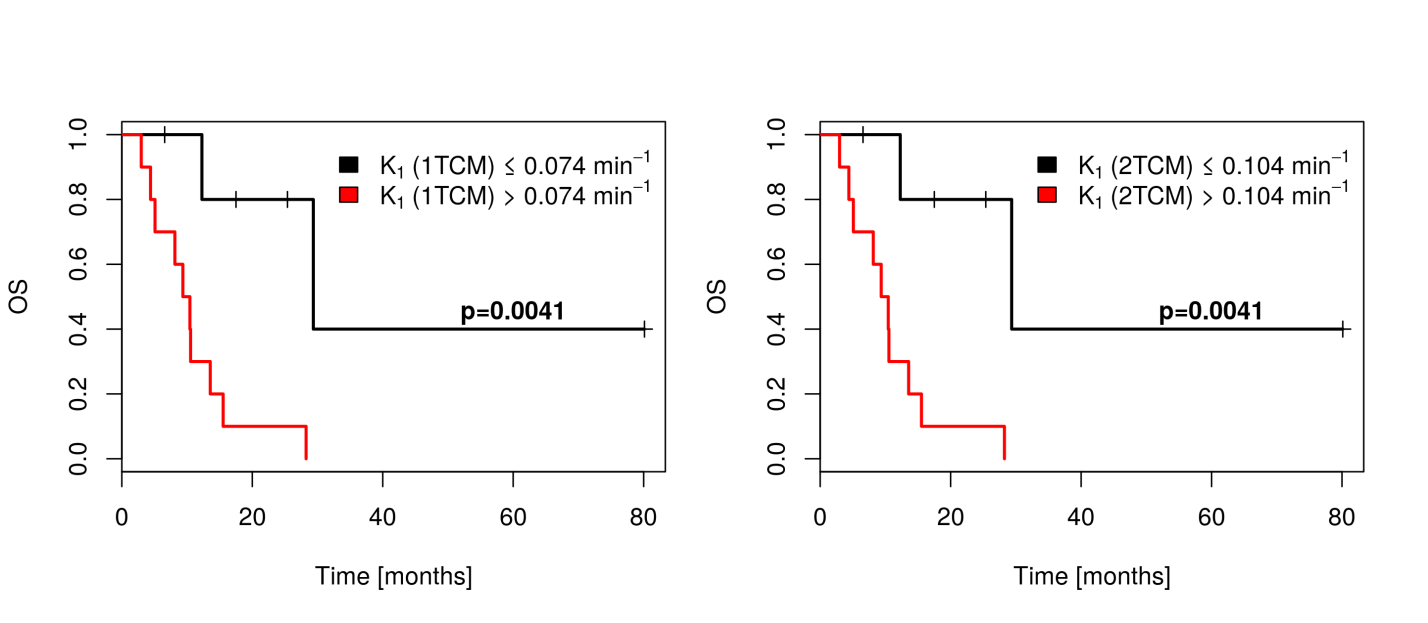


**Supplementary Figure S2**: Correlation of median parameter estimates with overall survival (OS). For both the 1TCM and 2TCM, K_1_ significantly separated patients into two groups with different overall survival (*p*=0.0041), with cutoffs of K_1_=0.074 min^-1^ and K_1_=.104 min^-1^, respectively.


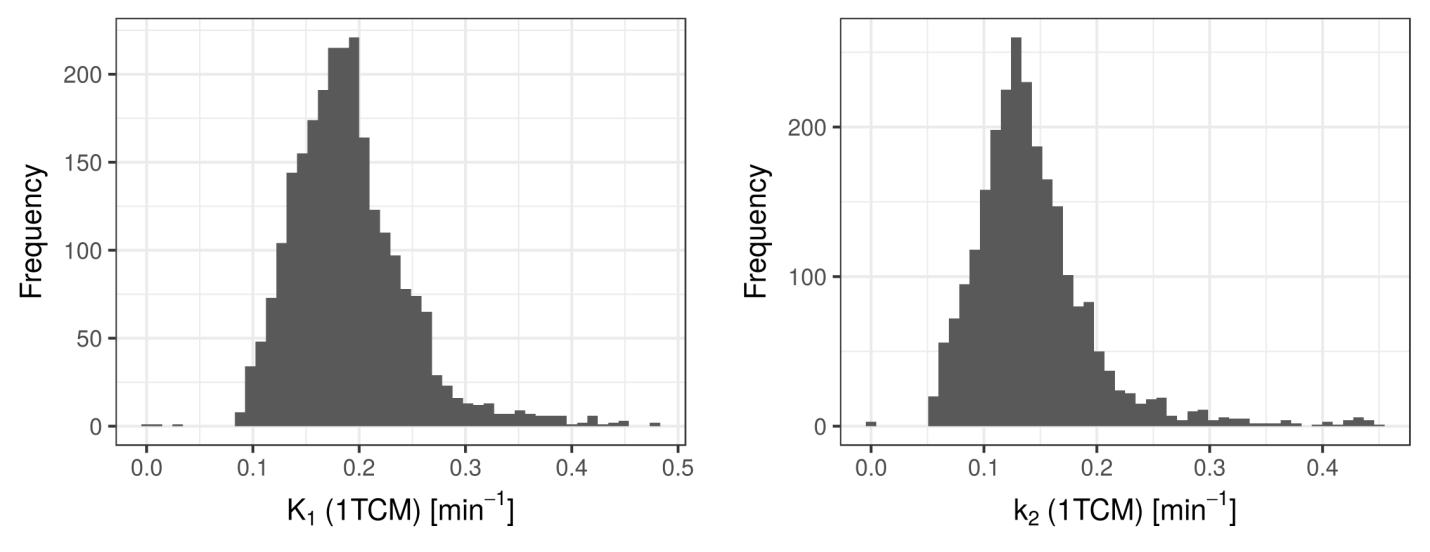


**Supplementary Figure S3**: Representative histograms of the distributions of parameter estimates K_1_ and k_2_ from fits with the 1TCM in an exemplary patient (same patient as in figure 6).
